# Protein-Rich Flours from Quinoa and Buckwheat Favourably Affect the Growth Parameters, Intestinal Microbial Activity and Plasma Lipid Profile of Rats

**DOI:** 10.3390/nu12092781

**Published:** 2020-09-11

**Authors:** Bartosz Fotschki, Jerzy Juśkiewicz, Adam Jurgoński, Ryszard Amarowicz, Paulina Opyd, Jürgen Bez, Isabel Muranyi, Iben Lykke Petersen, Moisés Laparra Llopis

**Affiliations:** 1 Division of Food Science, Institute of Animal Reproduction and Food Research, Tuwima 10, 10-748 Olsztyn, Poland; j.juskiewicz@pan.olsztyn.pl (J.J.); a.jurgonski@pan.olsztyn.pl (A.J.); r.amarowicz@pan.olsztyn.pl (R.A.); p.opyd@pan.olsztyn.pl (P.O.); 2 Fraunhofer Institute for Process Engineering and Packaging, Giggenhauser Str. 35, D-85354 Freising, Germany; juergen.bez@ivv.fraunhofer.de (J.B.); isabel.muranyi@ivv.fraunhofer.de (I.M.); 3 Department of Food Science, University of Copenhagen, 1958 Frederiksberg C., Denmark; ilp@food.ku.dk; 4 Molecular Immunonutrition Group, Madrid Institute for Advanced Studies in Food (IMDEA-Food), Ctra. de Canto Blanco n° 8, 28049 Madrid, Spain; moises.laparra@imdea.org

**Keywords:** quinoa, buckwheat, butyric acid, aryl hydrocarbon receptor, lipid profile, Wistar rats

## Abstract

In recent years, dietary products with quinoa and buckwheat have attracted attention mostly due to the high nutritive value of their protein fraction. However, their dietary effect on intestinal microbiota activity and related systemic responses are still poorly understood. Therefore, a 2 week study of twenty-eight growing male Wistar rats was conducted to investigate the effects of quinoa (QU) and buckwheat (BK) protein-rich flours on the growth parameters, intestinal microbial activity, plasma lipid profile, and inflammatory markers. The biological value of protein and body weight gain were considerably increased in the QU and BK groups compared with those in the soy protein isolate group. Moreover, both flours increased the microbial activity of α-glucosidase, β-glucosidase, and α-galactosidase and the concentration of short-chain fatty acids in the caecum. The studied flours favourably reduced the plasma total cholesterol and LDL cholesterol. In rats fed a diet with QU, elevated levels of plasma interleukin 6 and alanine transaminase were observed. The effect of QU on inflammatory markers may be related to the increased expression of aryl hydrocarbon receptor in the liver and to the decreased level of plasma albumin. In conclusion, quinoa and buckwheat protein-rich flours are valuable sources of proteins that favourably affect growth parameters, gut metabolism, and blood lipid profile in rats; however, only the buckwheat flour has no effect on inflammatory processes.

## 1. Introduction

In recent years, dietary products with quinoa (*Chenopodium quinoa*) and buckwheat (*Fagopyrum esculentum*) have become increasingly popular in the human diet. These pseudocereals have attracted attention mostly due to the high nutritive value of the protein fraction, as it provides all essential amino acids for human biological activity with values close to those set by the Food and Agriculture Organization (FAO); in addition, quinoa and buckwheat have excellent aminoacidic balance, as they are rich in sulphur-containing amino acids and lysine [1,2]. Furthermore, quinoa and buckwheat are characterized by the absence of gliadins (gluten-forming prolamins) and proteins corresponding to gliadins, which are considered toxic for celiac patients [3]. In addition to being a source of proteins, quinoa and buckwheat seeds are also a valuable source of carbohydrates, fat, vitamins, minerals, and phenols, which are associated with health benefits and some antinutrient compounds, such as saponins, phytic acid, tannins, and protease inhibitors [1,2].

The antinutrient compounds occurring in quinoa and buckwheat seeds, apart from their negative effects on consumers, may also activate health-promoting mechanisms, especially in the gastrointestinal tract. One example is phytic acid, which may exert beneficial effects on the large bowel environment similar to those associated with dietary fibre or oligosaccharides [4]. Additionally, the presence of tannins in the diet may considerably modulate intestinal microbiota activity and may exert favourable effects on intestinal morphology [5]. Furthermore, quinoa and buckwheat seeds may contain α-amylase-trypsin inhibitors (ATIs) [6]. These inhibitors are found in the endosperm of plant seeds, where they support natural defence against parasites and may regulate starch metabolism during seed development and germination [7]. As dietary compounds, ATIs are nutritional activators of innate immunity via the activation of Toll-like receptor 4 (TLR4). This receptor mostly mediates inflammatory mechanisms; however, the activation of TLR4 also induces anti-inflammatory processes through the stimulation of the tryptophan-metabolizing enzyme activity named indoleamine 2,3-dioxygenase and through the activation of aryl hydrocarbon receptor (AhR) [8]. The hepatic activation of AhR, which is a transcription factor, is also related to the regulation of lipid metabolism [9]. Recent studies on mouse and macrophage cells have shown that the immune response(s) induced by the administration of ATIs may differ and depend(s) on the type of cereal [10,11]. In addition to ATIs, trypsin inhibitors (rBTIs), which can also modulate immunometabolic mechanisms in hepatocytes, were also found in the protein fractions of buckwheat seeds [12]. Notably, recent reports have demonstrated the implication of innate immune effectors as determinants shaping gut microbiota and lipid homeostasis [13].

Most of the recent studies about quinoa and buckwheat seeds describe their chemical composition and potential health benefits. Nevertheless, with respect to the high nutritive value of their protein fractions, few nutritional studies have considered the effects of quinoa and buckwheat seeds on intestinal microbiota activity and related systemic responses. Therefore, the aim of this study was to investigate the effects of quinoa and buckwheat protein-rich flours on the growth parameters, intestinal microbial activity, and plasma immunometabolic markers of growing rats.

## 2. Materials and Methods

### 2.1. Preparation of Protein-Rich Flours from Quinoa and Buckwheat

Commercially available dehulled quinoa and buckwheat seeds were used for the preparation of protein-rich flours in pilot scale at Fraunhofer IVV, Freising, Germany. The seeds were obtained from Quinoa Marche srls, Italy, and Kümmel & Co. GmbH, Germany, respectively. Protein-rich flours were achieved by a three-step dry processing technique. First, the seeds were fragmented by impact milling with grinding gear based on the gear from the Ultraplex^®^ UPZ (Hosokawa Alpine AG, Augsburg, Germany). Then, seed fragments were classified by sieve classification in a sieve tower AS 200 (Retsch GmbH, Haan, Germany). The wheel speed (number of revolutions) of the impact mill was varied for each seed in order to optimize the process. For quinoa, the best protein enrichment was achieved by medium wheel speed (Level 2) prior to sieving (<710 µm-sieve), whereas for buckwheat, best results were obtained after fragmentation with high wheel speed (Level 1) prior to collecting the product between two sieve mesh sizes (>180 µm and <710 µm). The resulting sieve fractions were finally milled using an impact mill with 0.5 mm screen insert to obtain protein-rich quinoa and buckwheat flours with protein contents of 35.7% and 24.2%, respectively.

### 2.2. Chemical Composition of Protein-Rich Flours

For the determination of dry matter, the samples were dried to weight constancy at 105 °C in a thermos gravimetrical system (TGA 601, Leco Corporation, St. Joseph, MI, USA) according to the Association of Official Analytical Chemists method 925.10 [14]. The ash content was determined by ashing the samples in the oven at 550 °C until mass constancy had been achieved. The protein content was calculated based on the nitrogen content determined according to the Dumas combustion method [14] using a Nitrogen Analyzer FP 528 (Leco Corporation, St. Joseph, MI, USA). For quinoa a specific conversion factor of N × 5.85 was used [15] and for buckwheat N × 6.25 was used [16]. The protein was reported on a dry matter basis. The fat content was determined according to the Caviezel method [17]. Phenolic compounds were extracted from defatted flours with hexanes into 80% acetone (*v*/*v*). The extraction conditions were as follows: 3 × 30 min at 50 °C and a solid-to-solvent ratio of 1:10 (*w*/*v*) [18]. The extraction procedure was carried out in flasks in a shaking water bath (Julabo SW22, Julabo GmbH, Seelbach, Germany). Acetone from the combined extract was evaporated using a Büchi R-210 rotary evaporator. The sample was then freeze-dried. The content of total phenolics in the extracts was determined using Folin-Ciocalteu’s phenol reagent [19]. The results were expressed as (+)-catechin equivalents per g of flour. Condensed tannins were determined using the vanillin/HCl colorimetric method [20]. The results obtained were reported as (+)-catechin equivalents per g of flour. Phytic acid was determined using the assay kit from Megazyme (Cat. No. K-PHY). The activity of the trypsin inhibitors (ITAs) was determined according to Kakade et al. [21].

### 2.3. In Vivo Experiment

The animal protocol used in this study was approved by the Local Institutional Animal Care and Use Committee (Permission No. 34/2019; Olsztyn, Poland), and the study was performed in accordance with EU Directive 2010/63/EU for animal experiments. In the study, twenty eight male Wistar rats randomly allocated to four groups of seven rats each were used. The average of the initial body weight for all rats was 173.3 ± 1.1 g. All animals were housed individually over 2 weeks in metabolic cages (Tecniplast Spa, Buguggiate, Italy) with free access to water and semi-purified diets (detailed in Table 1). The diet intake was monitored in daily intervals. The environment was controlled with a 12 h light and 12 h dark cycle, a temperature of 21 ± 1 °C, and twenty air changes/h. With respect to the experimental feeding, a complete control diet (C) based on casein as the main protein source (supplemented with 0.2% DL-methionine), a second control diet (CS) based on commercial soya protein isolate (Edmir-Pol Co., Chorzow, Poland), and experimental diets with buckwheat protein-rich flour (BK) and quinoa protein-rich flour (QU) were used. The soya protein isolate consisted of 97.4% dry matter, 3.5% ash, 92.6% protein, 1% fat, and antinutrients; 1.3 mg/g phenolics, 14.1 mg/g phytic acid, and 10.6 IU/mg of trypsin inhibitor activity. All experimental diets were modified versions of the AIN-93G diet recommended by the American Institute of Nutrition [22]. To investigate the effects of protein fractions, all experimental diets had equal amounts of dietary protein. All physiological measurements were performed for each animal separately (n = 7 for each group).

### 2.4. Sample Collection and Basic Analyses

During the experiment, individual feed consumption, calorie intake, feed efficiency ratio, initial body weight, and body weight gain of rats were determined. In addition, the apparent biological value (BV) of protein was calculated by the following formula: BV = (N intake-N faecal-N urinal)/(N intake-N faecal) × 100. At the end of the experiment, the rats were weighed and anaesthetized, i.e., with ketamine (K) and xylazine (X) (K, 100/kg BW; X, 10 mg/kg BW), according to the recommendations for anaesthesia of experimental animals (Permission No. 34/2019; Olsztyn, Poland). After laparotomy, blood samples were collected from the caudal vein. Then, rats were sacrificed by cervical dislocation. This protocol allows to collect sufficient amount of plasma to perform all planned analyses. The plasma was prepared by centrifugation (350× *g*, 10 min, 4 °C) and kept frozen at −80 °C until assayed. Selected intestinal segments (caecum and colon) and internal organs (kidneys and liver) were removed and weighed. Liver weight as a percentage of the whole body weight (hepatosomatic index) was calculated. Liver samples were collected and stored at −80 °C until assayed. Samples of the caecal and colonic digesta were collected, and the pH was immediately measured using a microelectrode and a pH/ion meter (model 301; Hanna Instruments, Vila do Conde, Portugal).

In the fresh caecal digesta, the ammonia concentration was determined by the microdiffusion method in Conway’s dishes. After storage of the caecal digesta at −80 °C, the short-chain fatty-acid (SCFA) concentrations were measured using a gas chromatograph (Shimadzu GC-2010, Kyoto, Japan) in conjunction with a capillary column (SGE BP21, 30 m × 0.53 mm; SGE Europe Ltd., Milton Keynes, UK), as previously described [23]. In addition to the SCFA analysis, caecal fermentation processes were analysed based on the activities of selected bacterial enzymes (α- and β-glucosidase, α- and β-galactosidase, and β-glucuronidase), measured by the rate of release of ρ-nitrophenol (PNP) or o-nitrophenol (ONP) from the respective nitrophenyl glucosides, according to a previously described method [24].

Within the blood plasma, the following compounds were estimated using a biochemical analyser (Pentra C200, Horiba, Tokyo, Japan): triglycerides (TGs), total cholesterol (TC), fractions of high-density lipoprotein cholesterol (HDL), and low-density lipoprotein cholesterol (LDL), creatinine, urea acid, albumin, glucose, alkaline phosphatase (ALP), and aspartate and alanine aminotransferase activities (AST and ALT, respectively). To measure the concentration of interleukin 6 (IL-6) and interleukin 10 (IL-10) in the plasma, validated rat ELISA kits were used (Single Analyte ELISArray Kit, QIAGEN, Germantown, MD, USA).

### 2.5. RNA Isolation and Quantitative RT-PCR

Total RNA was extracted from liver samples using TRI Reagent (Sigma-Aldrich, Saint Louis, MO, USA), according to the manufacturer’s instructions. The quantity and quality of RNA were checked via a NanoDrop 1000 instrument (Thermo Scientific, Waltham, MA, USA) and by agarose gel electrophoresis. cDNA was synthesized from 500 ng of total RNA using a High-Capacity cDNA Reverse Transcription Kit with RNase Inhibitor (Applied Biosystem, Waltham, MA, USA). For the analysis of potential mechanisms regulating inflammatory processes and lipid homeostasis, aryl hydrocarbon receptor (AhR), peroxisome proliferator-activated receptor gamma (PPARγ), and Toll-like receptor 4 (TLR4) mRNA expression levels were measured using TaqMan^®^ Gene Expression Assays (Life Technologies, South San Francisco, CA, USA). Amplification was performed using a 7900HT Fast Real-Time PCR System under the following conditions: initial denaturation for 10 min at 95 °C followed by 40 cycles of 15 s at 95 °C and 1 min at 60 °C. Each run included a standard curve based on aliquots of pooled liver RNA. All samples were analysed in duplicate. The mRNA expression levels were normalized to those of the β-actin reference gene.

### 2.6. Statistical Analysis

The results are presented as the mean and standard error of the mean (SEM). The statistical analysis was conducted using one-way analysis of variance (ANOVA) or Kruskal–Wallis ANOVA by ranks, if the variance was unequal. Then, Duncan’s post hoc test or Dunn’s post hoc test with Bonferroni correction was used, depending on the ANOVA type, to check differences between individual groups. Differences were considered significant at *p* < 0.05. All calculations were made using STATISTICA software (StatSoft Corp., Krakow, Poland).

## 3. Results

The chemical composition of buckwheat and quinoa protein-rich flours is shown in Table 2. The dry matter content was 92.5% and 94.9%, whereas the ash content was 3.05% and 3.6% in the buckwheat and quinoa flour, respectively. The buckwheat flour consisted of 24.2% protein, whereas the quinoa flour consisted of 35.7% protein calculated on a dry matter (DM) basis. The buckwheat and quinoa flours were also rich in starch (53.6% and 20.5% DM, respectively) and other carbohydrates (11.3% and 24.0% DM, respectively). Moreover, the quinoa flour contained a significant amount of fat (14.7% DM), which was not the case for the buckwheat flour (3.4% DM). Moreover, both flours contained antinutrients, although their content in the buckwheat flour was much higher. This was partly due to the content of tannins, which were present only in the buckwheat flour (13.3 mg/g DM). However, the other phenolic and phytic acid contents were also higher in the buckwheat flour (7.41 and 15.9 mg/g DM, respectively) than in the quinoa flour (2.05 and 8.3 mg/g DM, respectively). Differences in the antinutrient contents were associated with a considerably higher activity of trypsin inhibitors (TIAs) in the buckwheat flour (13.3 IU/mg) than in the quinoa flour (0.4 IU/mg).

The growth parameters and basic indicators of the gastrointestinal function of rats fed experimental diets are shown in Table 3. After 14 days of experimental feeding, the body weight of rats decreased from the control diet containing soy protein isolate as the sole protein source (group CS) compared to that of the C group fed the standard casein diet. This decrease was prevented by the diet containing quinoa protein-rich flour (group QU) and especially by the diet containing buckwheat protein-rich flour (group BK). The lowest body weight gain occurred in the CS group, whereas both buckwheat- and quinoa-containing diets increased the body weight, and in the case of group BK, the increase was comparable to that of the C group. The diet and calorie intake decreased in the CS group, whereas in the BK and QU groups, it was comparable with that in the C group. In the CS group the feed efficiency ratio was the lowest, whereas in the BK and QU groups the values were comparable to that of the C group. The apparent biological value of protein (BV) was increased by dietary buckwheat flour to a level comparable with that of the C group. The liver weight decreased in all groups fed plant protein-containing diets. Thus, the hepatosomatic index of the animals receiving BK and QU flours showed a significant decrease compared to those that received control proteins (C and CS diets). The relative caecal tissue and digesta mass was increased by the BK and QU diets compared to the C diet. The pH value of the caecal digesta decreased in the BK and QU groups compared to both control groups. Collectively, these data suggest that BK and QU flours could lower liver fat accumulation and thereby limit the hepatic stress and inflammatory processes.

The activity of microbial enzymes in the caecal digesta was considerably altered by both dietary protein sources (Table 4). The α-glucosidase activity was almost two times higher in the BK and QU groups than in both control groups. The β-glucosidase activity was also higher in the BK and QU groups than in the CS group, but the increase was more pronounced and more than two times higher in the QU group. The α-galactosidase activity was more than two times higher in the BK and QU groups than in the CS group, whereas the β-galactosidase activity was significantly higher only in the QU group compared to the CS group. The β-glucuronidase activity increased in the CS and QU groups, whereas the diet containing buckwheat flour decreased this activity, but not to the level of the C group, where it was the lowest. These considerable changes in microbial activity only partly resulted in changes in caecal short-chain fatty-acid (SCFA) production (Table 4). Although the butyrate concentration in the caecal digesta increased in the BK and QU groups compared to both control groups, the acetate and total SCFA concentrations significantly increased only in the BK group compared to the CS group. These results reveal the positive effects of BK and QU that promotes the production of microbial-derived SCFA (i.e., butyrate, acetate, and propionate), which have been recognized as important substrates that improve epithelial barrier and hypoxia-inducible factor 1-alpha stabilization [25].

Given that the interaction between microbiota and their metabolites with distal gut are fundamental to maintain host’s health and fat partitioning [26], it was determined major parameters of lipid homeostasis (Table 5). The plasma concentrations of triglycerides, total cholesterol, and LDL cholesterol were lower in the CS group than in the C group. The triglyceride concentration did not differ between the CS group and the BK and QU groups. The total cholesterol and LDL cholesterol concentrations were lower in the BK and QU groups than in the CS group. The plasma ALT activity was significantly higher in the QU group than in the C and CS groups (Table 6). The plasma albumin concentration was higher in the CS and QU groups than in the C and BK groups. The plasma IL-6 concentration was comparable among groups fed plant protein-containing diets, and it was increased only in the QU group compared to that in the C group. These data indicate that plasmatic concentration of the pleiotropic IL-6 is not associated with the TIA (Table 2) either because of their poor bio-accessibility or susceptibility to digestive enzymes.

**Figure 1:**
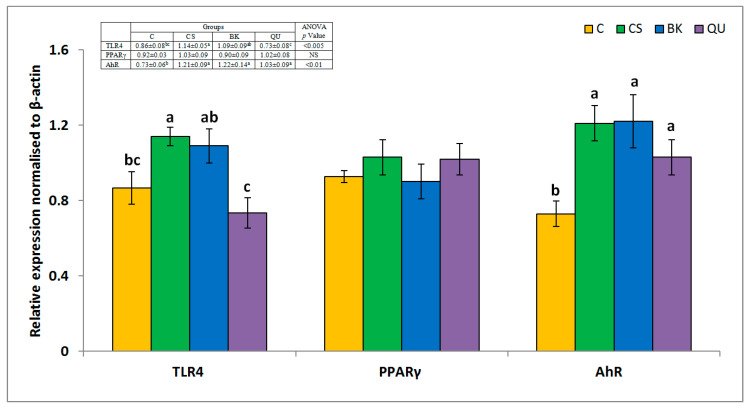
mRNA expression of selected factors related to immune–metabolic mechanisms in the livers of rats fed experimental diets. The values are the means ± SEMs. NS, *p* > 0.05. Mean values not sharing the same superscript letter within a row (a, b or c) are different at *p* < 0.05 in a post hoc test. C, rats fed a control diet with casein; CS, rats fed a diet with soy protein isolate; BK, rats fed a diet with buckwheat protein-rich flour; QU, rats fed a diet with quinoa protein-rich flour; AhR, aryl hydrocarbon receptor; PPARγ, peroxisome proliferator-activated receptor γ; TLR4, Toll-like receptor 4.

Changes in the hepatic expression of genes related to the activity of amylase/trypsin inhibitors or bacterial overgrowth (TLR4), lipid homeostasis (AhR), and nutrients fate (PPARγ) were determined to get insights into hepatic immunonutritional responses (Figure 1). Both TLR4 and AhR expression were increased in the CS group compared to that in the C group. The TLR4 expression was slightly and significantly decreased in the BK and QU group, respectively, compared to that in the CS group. AhR expression did not differ between the BK and QU groups and the CS group.

## 4. Discussion

Quinoa and buckwheat seeds are valuable sources of high-nutritional quality proteins that might even be comparable to casein from milk [1,27]. Indeed, in this study, the BV and growth parameters between rats fed diets with buckwheat protein-rich flour and with casein as a sole source of dietary proteins were similar. Interestingly, the mean growth parameters and BV values were lower in the group with commercial soy protein isolate than in the groups with the studied protein-rich flours. These changes might be attributed, at least in part, to differences in the bioavailability of amino acids and to the effects of antinutrients (i.e., saponins, protease inhibitors) on active nutrient transport at intestinal levels. Quinoa and buckwheat are rich sources of essential amino acids. In particular, methionine, lysine, arginine, tryptophan, and sulphur-containing amino acids are found at higher levels in the seeds of these species than in those of other cereals. Among all amino acids, the highest level of leucine is in quinoa seeds, whereas buckwheat seeds are the richest in phenylalanine [28]. Furthermore, phenolic compounds that were present in the examined flours can influence protein hydrolysis by their interactions with the protein substrate as well as protease itself [29]. Dietary phenolic compounds are also responsible for promoting growth and modulation of microbial activity in the gastrointestinal tract [30]. This particular feature of BK and QN flours may be a contributor, among other, to the increased bacterial glucosidase activity (Table 4).

Nutritional study on mice showed that dietary addition of whole grain quinoa flour favourably regulated the gut microbiota profile and that effect should be ascribed to polysaccharides and phenolic compounds [31]. The activity of the intestinal microbiota might also be modulated by dietary proteins and amino acids [32]. In a nutritional study on humans, Wu et al. (2011) [33] showed that dietary amino acid intake increases the relative abundance of Bacteroidetes. Members of this bacterial phylum are one of the major producers of β-galactosidase [34]. Indeed, both examined protein-rich flours considerably increased β-galactosidase activity in the caecum. These changes in enzymatic microbial activity might also be related to the growth/activity of the microbiota, which is essential for healthy functioning of the intestine and liver [35]. Undigested proteins and amino acids in the colon may also serve as an additional substrate for SCFA production [36]. Amino acids such as glycine, threonine, glutamate, lysine, ornithine, and aspartate can be metabolized by anaerobic bacteria to acetate; however, threonine, glutamate, and lysine can also be used for the synthesis of butyrate [37,38]. In this study, rats fed diets containing the studied flours exhibited considerably elevated microbial production of SCFAs, especially butyric acid, in the caecum. Moreover, when the buckwheat flour was added to the diet, an increased concentration of acetic acid in the caecum was observed. Butyric acid is the preferred energy source for colon epithelial cells, and together with the other SCFAs, it favourably decreases the pH and, thus, modulates the growth of microbiota in the hindgut [39]. These effects were also observed in the rats fed diets with quinoa and buckwheat protein-rich flours. In these groups, the weight of the caecal tissue considerably increased, whereas the pH value of the caecal digesta decreased. In addition, the examined flours were sources of carbohydrates including dietary fibre and resistant starch, which may also considerably affect intestinal microbiota activity. These compounds are efficiently metabolized to SCFAs by microbiota in the hindgut. The in vitro studies suggested that quinoa and buckwheat may have prebiotic effects, including enhanced growth of bacteria (e.g., *Bifidobacterium* spp., *Lactobacillus-Enterococcus*) responsible for the production of SCFAs [40,41].

The above changes in microbiota-derived SCFAs might be associated, to some extent, with the examined plasma lipid profile and markers of inflammation in rats [39]. Some researchers have linked higher concentrations of SCFAs in the hindgut with their potential inhibitory effects on the hepatic synthesis of cholesterol and, thus, on the decrease of plasma cholesterol [39,42]. Furthermore, the addition of quinoa and buckwheat flours to the diet reduced the total cholesterol, LDL cholesterol in the plasma, and the value of hepatosomatic index. Moreover, according to a recent study on the intestinal epithelial cell line, butyric acid is able to activate AhR [43] and, thus, negatively regulates several enzymes, including fatty-acid synthase and a cholesterol metabolism regulator in the form of sterol regulatory element-binding protein-1c [9]. In addition, the upregulated hepatic expression of AhR might also be a result of proteolytic microbial activity on tryptophan, where lactobacilli as well as members of the *Enterobacteriaceae* family play a major role. This microbial metabolism is concordant with the upregulation of AhR in the control group fed diet with soy protein isolate compared to the diet with casein, as consumption of soy diets has been found to antagonize AhR signalling [44]. Similarly, animals fed the QU and BK flour displayed an increased transcripts of AhR despite the different level of TIAs provided by both flours. In this context, elevated butyrate concentrations can also contribute to the downregulation of TLR4 trafficking to lipid rafts in the liver [45]. Given the role of AhR as a modulator of downstream TLR4 molecular signalling, the concurrent upregulation of both markers in liver tissue supports the increased production of tryptophan-derived metabolites as regulators of AhR and TLR4 transcripts.

Dietary protein intake seems to be responsible for the upregulation of TLR4, which could occur via the activation of the mTOR pathway, as transcription levels did not show correspondence with the different TIA levels in BK and QU diets. In this vein, the lower concentration of albumin was observed in the plasma of the QU group, which, as a carrier protein transporting metabolic products, regulatory mediators and nutrients, is also responsible for modulating inflammatory processes in the liver [46]. The fact that the QU diet increased the plasma concentration of IL-6 supports the implication of mTOR activation in stress signalling [47].

## 5. Conclusions

In the present feeding experiment, the addition of buckwheat and quinoa protein-rich flours to the diet favourably affected the growth parameters and gut metabolism of rats. However, between the examined flours, buckwheat exerted the strongest effects on BV and body weight gain. Both flours considerably increased microbial activity in the caecum; they increased the activity of α-glucosidase, β-glucosidase, and α-galactosidase as well as increased the production of SCFAs—mostly butyric acid—and decreased the digesta pH value. These changes might be associated with enhanced growth/activity of microbiota, which is important for the correct functioning of the gastrointestinal tract. Both the BK and QU diets positively affected the plasma lipid profile through a reduction of total cholesterol and LDL cholesterol. Among all the groups, only the diet of the CS group increased TLR4 expression in the liver of rats. Compared with the diet with casein, the QU diet increased the level of plasma inflammatory markers (IL-6 and ALT), which may be related to the higher hepatic expression of AhR than TLR4. Based on the obtained results, it can be concluded that the quinoa and buckwheat protein-rich flours are valuable sources of proteins that favourably affect the growth parameters, gut metabolism, and lipid profile. However, between the studied flours, only that from buckwheat seeds had no effect on inflammatory processes in rats. Nevertheless, this experiment was performed on healthy rats; therefore, to investigate inflammatory mechanisms regulated by examined protein-rich flours, further study on animal models of liver inflammation is needed.

## Figures and Tables

**Table 1:** Composition of the group-specific diets.

Ingredient (%)	Groups			
	C	CS	BK	QU
Casein	11.15			
D,L-methionine	0.20			
Soya protein isolate		10.8		
Buckwheat protein-rich flour			41.32	
Quinoa protein-rich flour				28.01
Cellulose ^1^	8	8	8	8
Soya oil	8	8	8	8
Mineral mix ^2^	3.5	3.5	3.5	3.5
Vitamin mix ^2^	1	1	1	1
Choline chloride	0.2	0.2	0.2	0.2
Cholesterol	0.3	0.3	0.3	0.3
Sucrose	5	5	5	5
Corn starch	62.65	63.2	32.68	45.99
Calculated calorie per kg of diet	3880	3880	3776	3986

All experimental diets were modified forms of the AIN-93G diet recommended by the American Institute of Nutrition. ^1^ The α-cellulose preparation was obtained from Sigma-Aldrich (No. C8002). ^2^ Recommended for the AIN-93G diet [22]. C, control diet with casein; CS, diet with soy protein isolate; BK, diet with buckwheat protein-rich flour; QU, diet with quinoa protein-rich flour

**Table 2:** Chemical composition of buckwheat and quinoa protein-rich flours.

	Buckwheat (BK)	Quinoa (QU)
DM (%)	92.5	94.9
Ash (%)	3.05	3.6
Protein (%)	24.2	35.7
Fat (%)	3.4	14.7
Carbohydrates ^a^ (%)	61.85	40.9
Antinutrients		
Tannins (mg/g)	13.9	0.0
Phenolics (mg/g)	7.41	2.05
Phytic acid (mg/g)	15.9	8.3
TIAs (IU/mg)	13.3	0.4

DM, dry matter; TIAs, trypsin inhibitors activity. ^a^ Carbohydrates = Dry matter − (Ash + Protein + Fat).

**Table 3:** Growth parameters and basic indicators of the gastrointestinal function of rats fed experimental diets.

Parameters	Groups				ANOVA *p* Value
	C	CS	BK	QU	
Diet intake (g/14 days)	258 ± 2.90 ^a^	231 ± 11.730 ^b^	265 ± 6.48 ^a^	242 ± 9.84 ^a,b^	<0.05
Calorie intake (kcal/14 days)	1001 ± 11 ^a^	896 ± 45 ^b^	999 ± 24 ^a^	964 ± 39 ^a,b^	<0.05
Feed efficiency ratio (g/g) *	0.32 ± 0.02 ^a^	0.15 ± 0.02 ^b^	0.26 ± 0.02 ^a^	0.25 ± 0.02 ^a^	<0.001
BV (%)	75.3 ± 2.64 ^a^	44.2 ± 2.20 ^c^	61.8 ± 0.538 ^a,b^	55.6 ± 0.901 ^b,c^	<0.001
Initial body weight (g)	170 ± 11.6	170 ± 11.9	169 ± 11.6	170 ± 11.9	NS
Final body weight (g)	253 ± 8.21 ^a^	206 ± 10.3 ^b^	238 ± 7.97 ^a^	232 ± 9.97 ^a,b^	0.01
Body weight gain (g)	83.5 ± 8.23 ^a^	35.4 ± 6.30 ^c^	68.7 ± 6.17 ^a,b^	61.8 ± 6.21 ^b^	<0.001
Liver (g/100 g BW)	4.79 ± 0.175 ^a^	3.68 ± 0.150 ^b^	4.00 ± 0.071 ^b^	3.79 ± 0.085 ^b^	<0.001
Hepatosomatic index (%) **	1.98 ± 0.11 ^a^	1.90 ± 0.08 ^a^	1.69 ± 0.06 ^b^	1.64 ± 0.07 ^b^	<0.05
Kidneys (g/100 g BW)	0.699 ± 0.019	0.746 ± 0.025	0.758 ± 0.015	0.741 ± 0.017	NS
Caecum					
Tissue (g/100 g BW)	0.195 ± 0.004 ^b^	0.262 ± 0.014 ^a^	0.261 ± 0.017 ^a^	0.314 ± 0.021 ^a^	0.001
Digesta (g/100 g BW)	0.614 ± 0.036 ^b^	0.828 ± 0.032 ^a^	0.861 ± 0.037 ^a^	0.777 ± 0.059 ^a^	0.005
Ammonia (mg/g)	0.244 ± 0.017	0.228 ± 0.028	0.216 ± 0.011	0.185 ± 0.008	NS
pH	7.69 ± 0.032 ^a^	7.56 ± 0.039 ^a^	7.38 ± 0.055 ^b^	7.28 ± 0.059 ^b^	<0.001
Colon					
Tissue (g/100 g BW)	0.376 ± 0.025	0.412 ± 0.010	0.373 ± 0.014	0.430 ± 0.017	0.072
Digesta (g/100 g BW)	0.443 ± 0.055 ^b^	0.671 ± 0.041 ^a^	0.490 ± 0.037 ^b^	0.470 ± 0.065 ^b^	<0.05
pH	7.80 ± 0.150	7.71 ± 0.134	7.41 ± 0.181	7.39 ± 0.118	NS

The values are the means ± SEMs. NS, *p* > 0.05. BV, apparent biological value of protein; C, rats fed control diet with casein; CS, rats fed diet with soy protein isolate; BK, rats fed diet with buckwheat protein-rich flour; QU, rats fed diet with quinoa protein-rich flour. * Diet intake per body weight gain. ** Liver weight as a percentage of the whole body weight. Mean values not sharing the same superscript letter within a row (a, b or c) are different at *p* < 0.05 in a post hoc test.

**Table 4:** Activity of microbial enzymes and the short-chain fatty-acid (SCFA) content in the caecum digesta of rats fed experimental diets.

Parameters	Groups				ANOVA *p* Value
	C	CS	BK	QU	
Microbial enzymatic activity (µmol/h/g digesta)					
α-Glucosidase	10.7 ± 0.620 ^b^	12.3 ± 1.32 ^b^	20.6 ± 1.72 ^a^	21.0 ± 2.36 ^a^	<0.001
β-Glucosidase	1.95 ± 0.253 ^d^	6.98 ± 0.749 ^c^	10.4 ± 0.926 ^b^	14.31.92 ^a^	<0.001
α-Galactosidase	7.61 ± 0.749 ^b^	10.9 ± 1.73 ^b^	24.9 ± 2.22 ^a^	29.6 ± 2.72 ^a^	<0.001
β-Galactosidase	21.2 ± 2.21 ^c^	29.6 ± 2.54 ^b,c^	39.7 ± 4.27 ^a,b^	60.0 ± 4.29 ^a^	<0.001
β-Glu curonidase	8.97 ± 1.05 ^c^	39.6 ± 5.87 ^a^	22.0 ± 2.54 ^b^	42.0 ± 3.82 ^a^	<0.001
SCFA, (μmol/g digesta)					
Acetate	37.9 ± 2.55 ^b^	43.0 ± 2.17 ^b^	58.0 ± 4.81 ^a^	47.7 ± 4.00 ^b^	<0.005
Propionate	8.42 ± 0.651	9.51 ± 0.562	10.3 ± 0.530	9.48 ± 0.217	NS
Isobutyrate	0.811 ± 0.059	0.941 ± 0.078	0.947 ± 0.070	0.890 ± 0.060	NS
Butyrate	6.49 ± 0.826 ^b^	7.14 ± 1.17 ^b^	12.2 ± 0.926 ^a^	12.1 ± 1.80 ^a^	<0.005
Isovalerate	0.922 ± 0.067	1.13 ± 0.116	1.06 ± 0.073	0.923 ± 0.091	NS
Valerate	0.887 ± 0.096	1.01 ± 0.061	0.889 ± 0.054	0.843 ± 0.052	NS
PSCFA	2.62 ± 0.214	3.08 ± 0.234	2.90 ± 0.180	2.66 ± 0.180	NS
SCFA total	55.4 ± 4.02 ^c^	62.7 ± 2.65 ^b,c^	83.4 ± 5.58 ^a^	71.9 ± 5.38 ^a,b^	<0.005

The values are the means ± SEMs. NS, *p* > 0.05. C, rats fed control diet with casein; CS, rats fed diet with soy protein isolate; BK, rats fed diet with buckwheat protein-rich flour; QU, rats fed diet with quinoa protein-rich flour. PSCFA; putrefactive short chain fatty acids (the sum of isobutyric, isovaleric and valeric acids). Mean values not sharing the same superscript letter within a row (a, b or c) are different at *p* < 0.05 in a post hoc test.

**Table 5:** Plasma lipid profile in rats fed experimental diets.

Parameters	Groups				ANOVA *p* Value
	C	CS	BK	QU	
TG (mmol/L)	3.16 ± 0.470 ^a^	1.25 ± 0.178 ^b^	1.68 ± 0.095 ^a,b^	1.38 ± 0.133 ^b^	<0.005
TC (mmol/L)	2.80 ± 0.111 ^a^	2.41 ± 0.106 ^b^	2.08 ± 0.103 ^c^	1.92 ± 0.097 ^c^	<0.001
HDL (mmol/L)	0.714 ± 0.027	0.906 ± 0.087	0.859 ± 0.107	0.894 ± 0.047	NS
LDL (mmol/L)	0.524 ± 0.035 ^a^	0.349 ± 0.040 ^b^	0.187 ± 0.022 ^c^	0.217 ± 0.021 ^c^	<0.001

The values are the means ± SEMs. NS, *p* > 0.05. HDL, high-density lipoprotein, LDL, low-density lipoprotein; TC, total cholesterol; TG, triacylglycerols. C, rats fed control diet with casein; CS, rats fed diet with soy protein isolate; BK, rats fed diet with buckwheat protein-rich flour; QU, rats, fed diet with quinoa protein-rich flour. Mean values not sharing the same superscript letter within a row (a, b or c) are different at *p* < 0.05 in a post hoc test.

**Table 6:** Inflammatory indicators in the plasma of rats fed experimental diets.

Parameters	Groups				ANOVA *p* Value
	C	CS	BK	QU	
AST (U/L)	63.5 ± 3.27	62.0 ± 0.933	61.4 ± 2.70	57.2 ± 2.33	NS
ALT (U/L)	18.9 ± 1.14 ^c^	20.5 ± 2.23 ^b,c^	24.6 ± 1.49 ^a,b^	26.4 ± 1.95 ^a^	<0.05
ALP (U/L)	310 ± 27.0 ^b^	408 ± 18.4 ^a^	367 ± 24.2 ^ab^	451 ± 40.8 ^a^	<0.05
Albumin (µmol/L)	400 ± 5.03 ^a^	371 ± 5.53 ^b^	388 ± 6.57 ^a^	367 ± 3.73 ^b^	0.001
Uric acid (µmol/L)	16.6 ± 1.94	20.7 ± 2.91	14.9 ± 1.84	16.0 ± 2.23	NS
Creatinine (µmol/L)	15.9 ± 2.51	17.4 ± 1.82	11.9 ± 1.61	14.4 ± 1.93	NS
IL-6 (pg/mL)	201 ± 1.50 ^b^	216 ± 8.00 ^a,b^	204 ± 5.55 ^a,b^	230 ± 7.54 ^a^	<0.05
IL-10 (pg/mL)	48.7 ± 2.18	48.5 ± 2.84	53.0 ± 2.71	54.6 ± 2.01	NS

The values are the means ± SEMs. NS, *p* > 0.05. ALT, alanine transaminase; AST, aspartate transaminase; ALP, alkaline phosphatase; IL-6, interleukin 6; IL-10, interleukin 10. C, rats fed control diet with casein; CS, rats fed diet with soy protein isolate; BK, rats fed diet with buckwheat protein-rich flour; QU, rats fed diet with quinoa protein-rich flour. Mean values not sharing the same superscript letter within a row (a, b or c) are different at *p* < 0.05 in a post hoc test.
